# Supplementary material for: The microscopic mechanism of water immersion and collapsibility in Malan loess with different particle size
Source: PLoS One. 2026 Apr 23;21(4):e0346775. doi: 10.1371/journal.pone.0346775 (PMC13105354; doi:10.1371/journal.pone.0346775)
Supplement: S1 Fig — (A) Undisturbed sandy loess; (B) Collapsible sandy loess; (C) Undisturbed silty loess; (D) Collapsible silty loess; (E) Undisturbed clayey loess; (F) Collapsible clayey loess. (PDF) [file pone.0346775.s001.pdf]

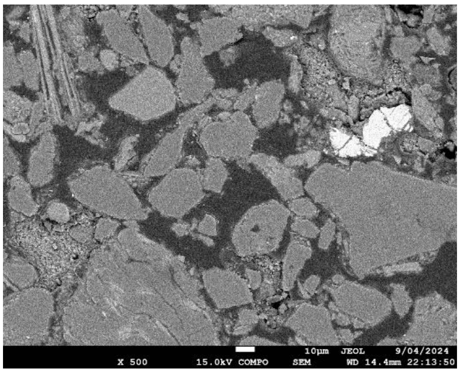

(A) Undisturbed sandy loess

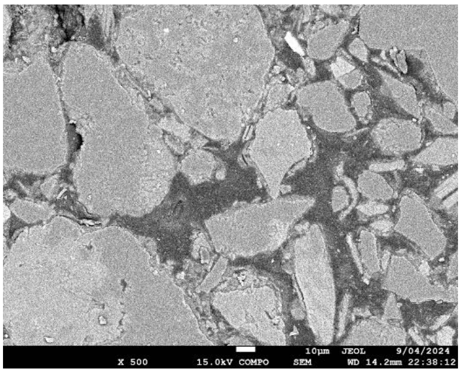

(B)Collapsible sandy loess;

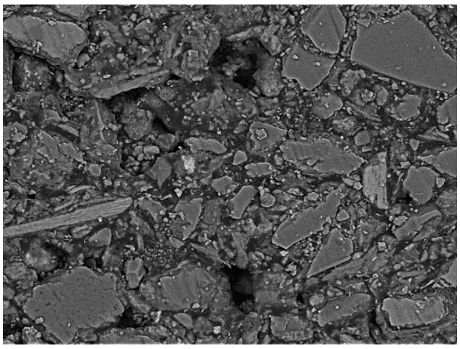

(C)Undisturbed silty loess

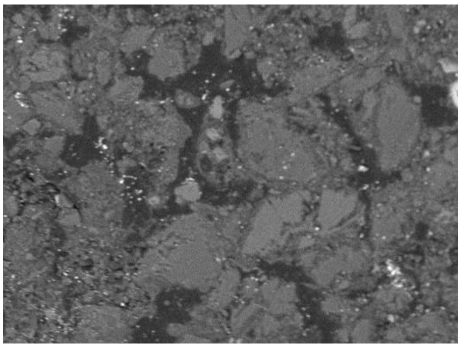

(D)Collapsible silty loess;

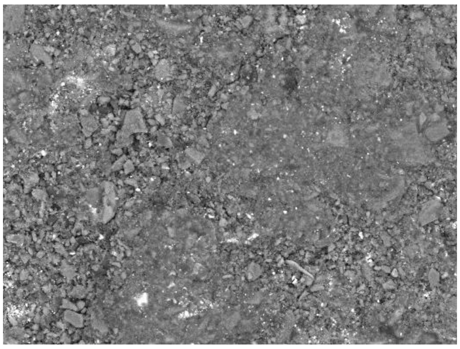

(E)Undisturbed clayey loess

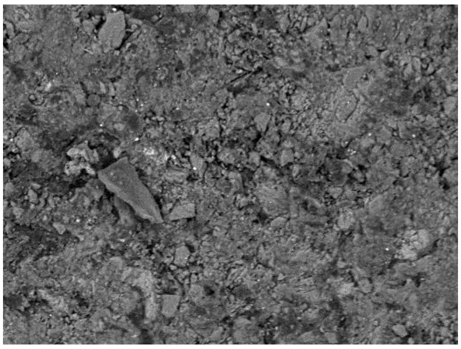

(F)Collapsible clayey loess.

S1 Fig. Raw picture of microstructure changes of Malan loess with different particle sizes. (A)Undisturbed sandy loess; (B)Collapsible sandy loess; (C)Undisturbed silty loess; (D)Collapsible silty loess; (E)Undisturbed clayey loess; (F)Collapsible clayey loess.
