# Supplementary material for: The microscopic mechanism of water immersion and collapsibility in Malan loess with different particle size
Source: PLoS One. 2026 Apr 23;21(4):e0346775. doi: 10.1371/journal.pone.0346775 (PMC13105354; doi:10.1371/journal.pone.0346775)
Supplement: Attachment — Submitted filename: Response to Reviewers.docx [file pone.0346775.s003.docx]

**Response to Reviewers**

Hello! We highly appreciate your valuable suggestions for improving the quality of this manuscript. In particular, we are grateful for your detailed and accurate revision comments — they have pointed out the deficiencies in the original manuscript and greatly inspired the authors. We have carefully revised the manuscript in accordance with the review comments, making the overall expression more standardized and the length more reasonable. We kindly request your review!

**Comments 1: Please ensure that your manuscript meets PLOS ONE's style requirements, including those for file naming.**

**Regarding Comment 1**: We have carefully revised the manuscript to fully comply with PLOS ONE's style requirements. The manuscript file has also been named strictly in accordance with the journal's specified format. Please check and confirm.

**Comments 2: Please note that PLOS One has specific guidelines on code sharing for submissions in which author-generated code underpins the findings in the manuscript. In these cases, we expect all author-generated code to be made available without restrictions upon publication of the work.**

**Regarding Comment 2**: We confirm that this manuscript does not involve any author-developed code. Therefore, the code sharing requirements specified in PLOS ONE's guidelines are not applicable to this study. We will fully cooperate with the journal's relevant review and publication processes.

**Comments 3: In your Methods section, please provide additional information regarding the permits you obtained for the work. Please ensure you have included the full name of the authority that approved the field site access and, if no permits were required, a brief statement explaining why.**

**Regarding Comment 3:** No specific permission was required for the fieldwork of this study. The research was conducted on non-private land and did not involve any protected areas. Additionally, the sample size was small and the sampling process was non-destructive, which did not require special approval in accordance with relevant national and local regulations. All field activities were carried out in compliance with academic ethics and legal requirements.

**Comments 4:** **Please state what role the funders took in the study. If the funders had no role, please state: "The funders had no role in study design, data collection and analysis, decision to publish, or preparation of the manuscript." If this statement is not correct you must amend it as needed. Please include this amended Role of Funder statement in your cover letter; we will change the online submission form on your behalf.**

**Regarding Comment 4:** Regarding the Role of Funder: The funders had no role in study design, data collection and analysis, decision to publish, or preparation of the manuscript.

**Comments 5: We note that [Figure 2 in your submission contain [map/satellite] images which may be copyrighted. All PLOS content is published under the Creative Commons Attribution License (CC BY 4.0), which means that the manuscript, images, and Supporting Information files will be freely available online, and any third party is permitted to access, download, copy, distribute, and use these materials in any way, even commercially, with proper attribution.**

**Regarding Comment 5:** We confirm that Figure 2 in the submission is an original hand-drawn image created by the authors. It does not involve any copyrighted materials from third parties, so there is no copyright infringement issue. The image fully complies with the requirements of PLOS ONE's Creative Commons Attribution License (CC BY 4.0) and can be freely accessed, downloaded, copied, distributed, and used by third parties with proper attribution.

**Comments 6: If the reviewer comments include a recommendation to cite specific previously published works, please review and evaluate these publications to determine whether they are relevant and should be cited. There is no requirement to cite these works unless the editor has indicated otherwise.**

**Regarding Comment 6:** We have carefully reviewed all the reviewer comments and confirmed that no specific publications are recommended for citation. The manuscript has strictly followed the journal’s citation guidelines, and all included citations are relevant to the core content of the study. We will maintain the current citation format and content in the revised manuscript.

**Comments 7: Sample Information: The "Materials" section mentions that sandy, silty, and clayey Malan loess samples were collected from Jingbian County, Yan'an City, and Jingyang County. However, the specific geological context of each sampling site is omitted. Elaborating on this background would greatly enhance the readers' understanding of the samples' representativeness.**

**Regarding Comment 7:** Regarding the Sample Information comment: We have comprehensively supplemented the specific geological background of each sampling site in the "Materials" section. As elaborated, the three sampling locations (Jingbian County, Yan'an City, and Jingyang County) are distributed across different regions of the Chinese Loess Plateau, with distinct zonal characteristics—from the northwest to the southeast, they correspond to sandy, silty, and clayey Malan loess respectively. For each site, we have added detailed descriptions including climate type, annual precipitation, geographical terrain (aeolian loess hilly region, loess ridge and gully region, loess tableland region), sedimentary environment, and particle composition characteristics (dominated by sand, silt, and clay respectively, as influenced by monsoon and regional geological conditions). This detailed supplementation fully clarifies the representativeness of each sample, helping readers better understand the correlation between sample properties and regional geological settings, in line with the journal’s requirements.

**Comments 8: Sampling Standardization: The description of sample preservation—encapsulation in PVC pipes—lacks critical details regarding the storage environment (e.g., controlled temperature and humidity ranges). These parameters are essential for assessing sample integrity and result reliability. Please supplement this information to ensure full traceability and standardization.**

**Regarding Comment 8:** Regarding the Sampling Standardization comment: We have comprehensively supplemented the critical details of sample preservation and storage environment as requested. The revised "Materials" section now includes the complete standardized process for sampling, encapsulation, storage, and transportation, as follows:

All samples were manually collected from a depth of 3–4 m to ensure uniform overlying pressure. To avoid damaging the loess structure, undisturbed soil blocks were first obtained via manual slotting, then cut and encapsulated in 110 mm-diameter PVC pipes—this method is key to preserving sample integrity during storage and transportation. After encapsulation, both ends of the PVC pipes were sealed with paraffin-coated cardboard and secured with tape; meanwhile, the sampling location, the upper/lower stratigraphic positions of the samples, and the sampling date were clearly labeled on the pipe surface. All gaps (including the joints between PVC pipes and paraffin-coated cardboard) were additionally wax-sealed to ensure airtightness.

For storage prior to laboratory testing, the PVC-encapsulated samples are placed in a controlled environment with a temperature range of 15–25°C (to prevent thermal expansion/contraction of the soil matrix) and a relative humidity (RH) of 40–60% (to avoid moisture absorption or desiccation of the undisturbed loess). During transportation, the undisturbed samples are protected from vibration, freezing (temperature <0°C), or excessive wetting (RH >70%); they are handled with care and prevented from inversion to avoid disrupting the stratigraphic order.

These supplementary details fully address the requirements for sample traceability and standardization, ensuring the integrity of the samples and the reliability of the research results.

**Comments 9: Experimental Process Details: In the "SEM Test" section, the protocol for sample preparation mentions a curing mixture of epoxy resin, acetone, ethylenediamine, and dibutyl phthalate, but omits the specific proportions of each component. Providing these key parameters is crucial for the reproducibility of the experiment.**

**Regarding Comment 9:** Regarding the Experimental Process Details comment: We have supplemented the specific proportions of the components in the curing mixture and elaborated on the complete protocol for the SEM test. The revised "SEM Test" section now includes detailed experimental procedures and key parameters to ensure reproducibility, as follows:

The following are the complete procedures for scanning electron microscopy (SEM) testing:(1) SEM Sample Preparation: To ensure the sample truly reflects the soil structure, the specimen was first preserved by freezing, followed by air-drying at a constant temperature of 20°C. After air-drying, a cylindrical soil unit with a diameter of 5 mm was extracted from the specimen and cured using a mixed curing agent. The components of the mixed curing agent were prepared in a mass ratio of epoxy resin: acetone: ethylenediamine: dibutyl phthalate = 100:200:7:2. The mixture was fully stirred uniformly before being used for soil sample curing.(2) Post-Curing Processing: After curing, the specimen was subjected to sequential cutting (adopting the sectional grinding method), grinding with 500-grit sandpaper, and polishing (reverse polishing for 6–8 minutes using a metallographic polisher) to produce a cylindrical thin section with dimensions of 5 mm (diameter) × 1 mm (thickness).(3) Gold Coating and Imaging: A gold film was sputtered onto the surface of the prepared cylindrical thin-section specimen to enhance its conductivity. The SEM test was conducted promptly (within 30 minutes) after gold coating to prevent oxidation of the gold film and surface contamination from air exposure.

These supplementary details, especially the specific proportions of the curing mixture components, fully address the requirement for experimental reproducibility.

**Comments 10: Chart Clarity: The captions for figures, such as Figure 4 ("Loest Particle Size Distribution") and Figure 5 ("Mineral Test Results of Loess with Different Clay Contents"), should explicitly state the correspondence between sample numbers and their respective sampling locations. This addition is necessary to allow readers to easily identify the source of each data presentation.**

**Regarding Comment 10:** We have fully revised the captions of Figure 4 and Figure 5 to explicitly state the correspondence between sample types, sampling locations, and regional contexts. The standardized and corrected captions are as follows:

Fig. 4. Particle size distribution of loess. (A) Sandy loess (Jingbian County, northwestern Chinese Loess Plateau); (B) Silty loess (Yan'an City, central Chinese Loess Plateau); (C) Clayey loess (Jingyang County, southern Chinese Loess Plateau).

Fig. 5. Test results of loess minerals with different clay contents. (A) Jingbian sandy loess (Jingbian County, northwestern Chinese Loess Plateau): A-Quartz; B-Albite; C-Muscovite; D-Calcite; (B) Yan'an silty loess (Yan'an City, central Chinese Loess Plateau): A-Quartz; B-Albite; C-Muscovite; D-Calcite; (C) Jingyang clayey loess (Jingyang County, southern Chinese Loess Plateau): A-Quartz; B-Albite; C-Muscovite; D-Calcite; E-Kaolinite.

**Comments 11:** Conclusion Specificity: The fourth point of the conclusion section, which proposes a "micro-collapse mechanism model," is currently too vague. To strengthen the impact and practicality of this finding, please elaborate on the model's core elements (e.g., particle contact modes, pore evolution laws) and its specific applications in fields such as loess region engineering geology and disaster prevention.

**Regarding Comment 11:** To address the comment on Conclusion Specificity, we have comprehensively refined the "micro-collapse mechanism model"—now explicitly defined as the "particle-pore-clay synergistic collapsibility mechanism model"—by explicitly clarifying its core components, supplementing specific quantitative and qualitative characteristics to enhance operability, and detailing practical application scenarios with concrete engineering cases, thereby eliminating vagueness in the original version and strengthening the finding’s impact and relevance.

Specific revisions are as follows: Based on the microstructure, parameter changes, and sedimentary structures of Malan loess pre- and post-collapsibility, a particle-pore-clay synergistic collapsibility mechanism model was established. Its core elements: pre-collapsibility, coarse particles are in point-point contact with dispersed clay filling pores; post-collapsibility, contact shifts to point-surface/surface-surface, with clay migrating via water to form a thin cementation layer at contacts. Pores undergo three-stage transformation (macropore reduction, mesopore expansion, micropore reconstruction), making the pore structure shift from extremely heterogeneous to relatively homogeneous. Clay content dictates collapsibility sensitivity: sandy loess (low clay) collapses mainly via pore collapse, clayey loess (high clay) via inter-particle cementation layer destruction. This model applies to loess engineering geology and disaster prevention, guiding foundation pre-treatment, assisting slope collapsible disaster prediction, and optimizing underground structure stability measures.
