# Supplementary material for: The microscopic mechanism of water immersion and collapsibility in Malan loess with different particle size
Source: PLoS One. 2026 Apr 23;21(4):e0346775. doi: 10.1371/journal.pone.0346775 (PMC13105354; doi:10.1371/journal.pone.0346775)
Supplement: Attachment — Submitted filename: Response_to_Reviewers_auresp_2.docx [file pone.0346775.s004.docx]

**Response to Reviewers**

Hello! We highly appreciate your valuable suggestions for improving the quality of this manuscript. In particular, we are grateful for your detailed and accurate revision comments — they have pointed out the deficiencies in the original manuscript and greatly inspired the authors. We have carefully revised the manuscript in accordance with the review comments, making the overall expression more standardized and the length more reasonable. We kindly request your review!

**Comments 1: The title "Research on the microscopic mechanism of water immersion and collapsibility in Malan loess with different particle size" contains redundant wording "Research on". It is recommended to delete "Research on".**

**Regarding Comments 1:** We thank the reviewers for their valuable comments. As suggested, the redundant term "Research on" in the title has been deleted. The revised English title is *Microscopic Mechanism of Water Immersion Collapse of Malan Loess with Different Particle Sizes*, while the Chinese title remains unchanged, making the title more concise, standardized and closely aligned with the research theme.

**Comments 2: It is recommended to improve the clarity of all figures. Furthermore, there are inconsistencies: the sequence of Figure 1 (B) and (C) appears to be reversed; the content presented in Figure 6 (A) and (B) is inconsistent; Figures 7 and 8 are discontinuous. The authors are advised to carefully check all figures and their correspondence to the text.**

**Regarding Comments** 2: We thank the reviewers for their valuable comments. Regarding the issues with figures and tables, we have made targeted revisions and a full check: (1) All figures and tables have been optimized to high resolution for improved clarity and detail discernibility; (2) The layout order of subplots (B) and (C) in Fig. 1 and the content correspondence of subplots (A) and (B) in Fig. 6 have been corrected for information consistency; (3) The numbering of Figs. 7 and 8 has been standardized with missing numbers supplemented for continuous numbering. We have also fully verified the consistency between all figures/tables and their citations in the main text, ensuring complete match of numbering, content and descriptions. All revisions have been made at the corresponding positions in the manuscript.

**Comments 3: It is recommended to provide details on how the physico-mechanical parameters in Table 1 were obtained (e.g., test standards, calculation methods).**

**Regarding** **Comments 3:** We thank the reviewers for their valuable comments. Regarding the missing determination methods for physical and mechanical parameters in Table 1, we have supplemented all relevant details: a note on parameter testing methods has been added above Table 1, clearly stating the corresponding test standards, specific methods and calculation basis for each index. We have verified the accuracy of the supplemented content to ensure full consistency between the testing methods, standards and the parameters in the table, thus clarifying the derivation logic of all parameters. All supplements have been completed at the corresponding position in the manuscript.

**Comments 4: In line 180, "initial height Ho" refers to the sample's initial height before loading. In line 191, "H0" is used again for the sample height after stabilization under each loading stage. To avoid confusion, it is suggested to use distinct notations.**

**Regarding Comments 4:** We thank the reviewers for their valuable comments. Regarding the inconsistent use of symbols in the manuscript, revisions have been made as suggested: the symbol H_0_ (representing the initial height of specimens before loading) at Line 180 has been adjusted to H, which is clearly distinguished from H_0_ (denoting the stable height of specimens under various load levels) at Line 191, thus completely eliminating symbol confusion. We have also fully checked the consistency of all symbol usages throughout the manuscript to ensure clear and unambiguous designation of all physical quantity symbols. All revisions have been made at the corresponding positions in the manuscript.

**Comments 5: The authors use formula (1) to calculate the collapsibility coefficient . Please clarify why this specific method was chosen. Is it based on a specific standard or theoretical framework? Supplementary explanation is needed.**

**Regarding Comments 5:** We thank the reviewers for their valuable comments. Regarding the basis for selecting the calculation method of the collapse coefficient, we have added a clear explanation: the collapse coefficient in this paper is calculated using Eq. (1), which strictly complies with the definition requirements for the collapse coefficient specified in the Chinese national technical standard *Code for Building Construction in Collapsible Loess Regions* (GB 50025-2018). This standard takes the collapse coefficient δ_s_ as the core index for evaluating loess collapsibility, providing a direct basis for the classification of loess collapsibility grades and geotechnical engineering design in loess regions. The adoption of this calculation method in this paper ensures the consistency and standardization of test data with engineering practice. Relevant explanations have been supplemented and completed at the corresponding position in the manuscript.

**Comments 6: In the SEM Test section, details were added per reviewer suggestions. However, some aspects require further clarification: In the line 206, what is the control criterion for "fully stirred uniformly" of the mixed curing agent? Also, specify the curing time and environmental conditions (temperature, humidity) for the soil sample during the curing process.**

**Regarding Comments 6:** We thank the reviewers for their valuable comments. Regarding the detailed issues in the SEM test section, we have supplemented and clarified the relevant control criteria and curing conditions: at Line 206, the judgment criterion for the curing agent mixture being "thoroughly stirred uniformly" is defined as forming a homogeneous, transparent viscous liquid with no obvious delamination, particle agglomeration or local sedimentation, and a consistent color; the mixture can only be used for soil sample curing when this criterion is met. Meanwhile, it is specified that the soil samples mixed with the curing agent are cured under the same environmental conditions as in the air-drying stage. The descriptions of the relevant control criteria and curing conditions have been supplemented and completed at the corresponding positions in the manuscript.

**Comments 7: In the PCAS Test section, regarding the quantitative pore analysis of loess SEM images, how were the specific samples for analysis selected? Please justify how these analyzed samples are representative of the entire soil type**

**Regarding Comments 7:** We thank the reviewers for their valuable comments. Regarding the sampling basis and representativeness of specimens in the Pore Structure Analysis System (PCAS) tests, we have supplemented and clarified the relevant justifications: prior to the quantitative pore analysis of loess SEM images, strict specimen selection criteria were formulated to ensure the reliability and representativeness of the analysis results, specifically: (1) Sampling and sample preparation were prioritized in homogeneous soil regions to avoid the interference of local structural anomalies on the statistical results of pore parameters; (2) At least 3 parallel specimens were prepared for each test group, and non-repetitive fields of view were randomly selected from each specimen for imaging analysis to reduce accidental errors caused by a single specimen or field of view. The micro-pore structural characteristics of the specimens selected in accordance with the above criteria can reflect the overall structural properties of the corresponding soil to a certain extent. The relevant explanations on specimen sampling basis and representativeness have been supplemented and completed at the corresponding positions in the manuscript.

**Comments 8: Line 254 states that "All samples exhibit a particle size range of 0.1-100 ". However, the particle size distribution curves in Figure 4 for all three loess types extend beyond 100 . The authors should check and clarify this apparent contradiction.**

**Regarding Comments 8:** We thank the reviewers for pointing out this inconsistency, which upon verification stems from a descriptive oversight and is now clarified and corrected as follows: the inaccurate statement at Line 254 that "the particle size range of all specimens is 0.1~100 μm" actually refers only to the silt and clay particle fraction of the specimens, while the full particle size range of the loess specimens tested in this study is 0.1~200 μm (including a small amount of fine sand particles); the particle size distribution curves of the three loess types in Fig. 4 fully reflect the full particle size composition characteristics of the specimens, with the fraction larger than 100 μm being fine sand particles accounting for less than 5% of the total mass, and the discrepancy between the textual description and the figure arose because the main text focused on the dominant silt-clay fraction due to the extremely low content of fine sand, with relevant revisions completed at the corresponding position in the manuscript.

**Comments 9: In line 257, "coarse-grained particles" refers to "fine sand and coarse silt". In line 265, "fine-grained particles" refers to "clay and fine silt". These descriptions are somewhat vague. Please provide specific size ranges for these particle categories**

**Regarding** **Comments 9:** We thank the reviewers for their valuable comments. Regarding the ambiguous classification of coarse and fine particle sizes, we have supplemented the specific particle size ranges in the manuscript and revised the particle characteristic descriptions accordingly for consistent definitions aligned with the general standards for engineering geological research on loess: "coarse particles (fine sand, coarse silt)" at Line 257 are defined as fine sand (100~200 μm) and coarse silt (50~100 μm), and "fine particles (clay, fine silt)" at Line 265 as clay (< 5 μm) and fine silt (5~20 μm). We have also revised the description of the peak value of the particle frequency curve for clayey loess. All relevant content has been revised and improved at the corresponding positions to ensure clear particle size classification and accurate data description.

**Comments 10: Line 265 states that the frequency curve peak for clayey loess is at "<20 ", yet Figure 4(C) suggests a peak at a size >20 . The authors should verify and correct this discrepancy.**

**Regarding** **Comments 10:** We thank the reviewers for their careful comments. Upon verification, this discrepancy arose from an oversight in the description, and we now clarify and correct it as follows: (1) Rechecking the particle size frequency curve of clayey loess in Fig. 4(C) confirms that its peak particle size is actually 25 μm, which falls within the fine silt range (5~50 μm) and is consistent with the overall characteristic of clayey loess being dominated by fine particles; (2) The inaccurate statement at Line 265 that "the peak is in the range of <20 μm" has been revised to "the frequency curve peaks in the range of 20~30 μm".

**Comments 11: In the Collapsibility deformation characteristics section, assuming H0 is the initial sample height, the observation that the collapsibility coefficient first increases and then decreases with increasing axial pressure implies, according to formula (1), that the axial deformation itself also first increases and then decreases with pressure. If this is indeed the case, it would be a notable finding. The authors should provide a clear physical explanation for this phenomenon.**

**Regarding** **Comments 11:** We thank the reviewers for their careful comments. Regarding the axial deformation law reflected by the collapsibility coefficient increasing first and then decreasing with rising axial pressure and its physical mechanism, we have supplemented a systematic interpretation and mechanistic analysis in the collapsible deformation characteristics section: we clearly define this trend as a typical mechanical response of collapsible loess under graded loading, with the axial collapse deformation showing a synchronous increasing-then-decreasing variation with axial pressure. Combined with the microstructural traits of loess, we elaborate on its intrinsic physical mechanism in two stages (low-to-medium axial pressure < 600 kPa and high axial pressure > 600 kPa) from the perspective of the coupling effect of soil skeleton compaction and water-induced cementation degradation. We also supplement key data including the peak collapse pressure and collapsibility differences among various loess types to improve the scientificity and completeness of the mechanistic interpretation. Relevant analytical content has been supplemented and refined at the corresponding positions in the manuscript, clearly illustrating the formation mechanism and engineering significance of this phenomenon.

**Comments 12: In lines 192-193, the collapsibility test procedure involves saturating the sample by immersion after stabilization under the final load stage. As noted in the particle size analysis, clayey loess is dominated by fine particles. The high fine particle content can significantly affect the sample's permeability and the collapse during immersion. The authors could refer to studies like "DOI: 10.1007/s10064-025-04350-8" for a microscopic perspective on such effects.**

**Regarding** **Comments 12:** We thank the reviewers for their valuable suggestions. Regarding the micro-mechanism by which the high fine particle content of clayey loess affects its permeability and immersion collapse process, we have supplemented the relevant analysis after the collapse test procedure at Lines 192-193 with reference to the related study [DOI: 10.1007/s10064-025-04350-8] as suggested. We elaborated on the microcosmic effects of fine particles on the permeability and immersion collapse of clayey loess from two aspects: fine particles filling pores to form a dense micropore network, and surface adsorption of clay minerals enhancing particle cementation. The inhibitory effects of these processes on the soil water infiltration rate and cementation softening process, as well as their impacts on the initiation time and deformation development rate of immersion collapse, were clarified. Meanwhile, the actual effect of this micro-mechanism on the collapse test results was explained in combination with the test procedure. Relevant analytical content has been supplemented and refined at the corresponding position in the manuscript, improving the interpretation of the correlation between fine particle effects and the collapse test process.

**Comments 13: In lines 363-365, the authors attribute the decrease in particle fractal dimension after collapse to changes in particle contact modes (e.g., point-to-point to point-to-edge), which simplifies particle boundaries. To better discuss the influence of particle morphology on particle crushing and subsequent changes in contact state, the authors could refer to relevant literature, such as: DOI: 10.1016/j.powtec.2024.120592 and DOI: 10.1016/j.powtec.2023.119204.**

**Regarding Comments 13:** We thank the reviewers for their valuable comments! In response to the comments on the collapse test procedure and the micro-scale influence mechanism of fine particles in clayey loess, we have supplemented and improved Lines 192–193 of the original text and the subsequent analysis sections by combining the particle size analysis results and the recommended references. The specific revisions are as follows:

The parameter that the content of fine particles (<20 μm) in the clayey loess used in this study accounts for 59% is added, clarifying its core regulatory effect on the permeability and water immersion collapse of the specimens, and establishing the correlation between the test operation and the microstructural characteristics of the material.

Drawing on the recommended reference (DOI: 10.1007/s10064-025-04350-8), the dual regulatory mechanism of fine particles on loess collapse is newly added. The differential effects of fine particles are elaborated in combination with the medium-low axial pressure stage (<600 kPa) and high axial pressure stage (>600 kPa), and the logical chain of *"microstructural characteristics of fine particles → permeability variation → cementation state evolution → macroscopic collapse response"* is constructed.

The above reference is standardizedly cited. The test procedure, particle size analysis, micro-scale mechanism and macroscopic collapse laws are systematically integrated to realize the correspondence among test phenomena, material characteristics and intrinsic mechanisms, thus improving the scientificity and completeness of the analysis.

**Comments 14: The authors propose a "particle-pore-clay synergistic collapsibility mechanism model". This model essentially describes structural changes in Malan loess before and after collapse. How does this model differ from existing collapse theories, particularly the structural theory mentioned earlier? A comparative analysis is recommended. Furthermore, for future work, analyzing collapse from a material damage perspective could be insightful. The authors may refer to: DOI: 10.1016/j.ijrmms.2024.106014.**

**Regarding** **Comments 14:** We sincerely appreciate the reviewer’s insightful comments and constructive suggestions. In response to the request for clarifying the differences between the proposed "particle-pore-clay synergistic collapsibility mechanism model" and existing collapsibility theories (especially the structural theory mentioned earlier), we have supplemented a detailed comparative analysis in the revised manuscript. Meanwhile, we highly agree with the suggestion of analyzing the loess collapsibility process from the perspective of material damage, and the recommended reference (DOI: 10.1016/j.ijrmms.2024.106014) has been thoroughly studied and cited as an important reference for our future research.

**Comments 15: Regarding engineering applications, the statement that the model "applies to loess engineering geology and disaster prevention, guiding foundation pre-treatment, assisting slope collapsible disaster prediction, and optimizing underground structure stability measures" is rather general. The authors should elaborate on how it can be specifically applied. For example, the authors may refer to DOI: 10.1016/j.tust.2025.107144, which first identifies influencing factors on construction conditions and then proposes specific application measures for given scenarios.**

**Regarding** **Comments 15:** We appreciate your valuable comments! Your suggestion that the engineering application section is overly general and needs specific scenario elaboration is highly pertinent and crucial for enhancing the research’s practical guiding value. We have carefully studied the recommended sample paper (DOI: 10.1016/j.tust.2025.107144) and drawn on its reasoning logic of "identifying key influencing factors — proposing targeted control measures". Specific revisions:

Closely adhering to the "particle-pore-clay synergistic collapsibility mechanism model", we clarify the dominant influencing factors (particle contact transformation, three-stage pore evolution, core role of clay content) at the beginning, corresponding to the sample paper’s "clarifying working condition factors" step.

Decomposing the original general description into three typical engineering scenarios (foundation pretreatment, slope collapsibility prediction/early warning, underground structure stability optimization), with targeted solutions based on the model (e.g., dynamic compaction/compaction piles for sandy loess foundations, pre-soaking/chemical improvement for clayey loess foundations).

Transforming the section from "principle-based guidance" to "mechanism-based targeted suggestions", clarifying the connection between micro-mechanism research and practical engineering solutions, and enhancing the paper’s practicality and persuasiveness.

The revised content has been integrated into the manuscript. We sincerely thank you again for your careful review and constructive comments, which have greatly helped improve this paper.

**Comments 16: Although the manuscript proposes a “generalized collapse mechanism model,” the novelty and scientific contribution of this model remain unclear. Much of the mechanism discussion reiterates well-established structural interpretations of loess collapsibility. The authors should clearly articulate how their findings advance beyond existing theories and models in the literature, and specify what new insight is gained from the comparison of particle-size groups beyond descriptive differences.**

**Regarding** **Comments 16:** We sincerely appreciate the reviewer’s comments. To address the unclear innovation of the model and repetitive mechanism analysis, we have supplemented the theoretical breakthroughs of the model and innovative insights from particle size grouping research in the revised manuscript, with key revisions as follows:

1. The proposed "particle-pore-clay synergistic collapsibility mechanism model" breaks through the single-factor limitation of existing theories by constructing a coupled dynamic system of genetic endowment, clay physicochemical behaviors and pore evolution, revealing the synergistic interaction mechanism among three factors and establishing quantitative micro-macro correlations, which is a substantive breakthrough from the qualitative analysis of traditional theories.
2. Beyond descriptive disparity analysis, the particle size grouping comparison yields innovative cognitions: (1) Particle size distribution is the intrinsic link between genetic genesis and collapsibility; (2) Different loess types have differentiated synergistic collapse mechanisms, clarifying the microscopic essence of graded collapsibility of loess with different particle size characteristics.
3. We have deleted redundant repetitive descriptions of existing structural theory in the mechanism analysis section, added a dedicated part to highlight the model’s theoretical breakthroughs and the study’s innovative cognitions, and optimized the logical expression to fully reflect the academic contributions of this study.
4. The revised content has been integrated into the mechanism analysis and model discussion section (Lines 468-509) of the manuscript.

**Comments 17: The study relies on single sampling sites for each loess type, and the manuscript does not provide information on sample replication, intra-site variability, or experimental reproducibility. Without statistical measures (e.g., variance, confidence ranges, repeat test results), it is difficult to assess the reliability and regional representativeness of the findings.**

**Regarding** **Comments 17:** We appreciate the rigorous comments from the reviewers! Regarding the issues of sample repeatability, internal variability at sampling sites, experimental reproducibility and supplementation of statistical indicators, we have made systematic improvements with the specific revisions as follows: The original single sampling site design for each loess type has been optimized. Three parallel sub-sampling sites were added to each of the three main sampling sites (Jingbian for sandy loess, Yan'an for silty loess, Jingyang for clayey loess), resulting in a total of 9 undisturbed soil samples (3 loess types × 3 parallel sub-samples for each type). All samples were collected from the same Late Pleistocene (Q₃) Malan loess layer at a depth of 3~4 m to avoid interference from stratigraphic differences. Preliminary field tests showed that the relative deviation of natural water content among sub-samples at the same sampling site was ＜3% and that of natural density was ＜2%, which verified low internal variability at the sampling sites and ensured the good representativeness and reliability of the samples.

**Comments 18: The SEM observations and PCAS results are interesting, but the discussion remains largely qualitative. There is no analysis linking microstructural parameters to collapsibility outcomes (e.g., correlation, regression, sensitivity analysis). Similarly, fractal analysis is presented descriptively without deeper interpretation. Strengthening microstructure–mechanism–deformation connections would greatly improve scientific rigor.**

**Regarding** **Comments 18:** Thank you for the reviewers' comments! To address the issues that the test results of Scanning Electron Microscopy (SEM) and Particle and Crack Image Recognition and Analysis System (PCAS) were mostly qualitative, there was a lack of correlation between microstructural parameters and collapsibility indicators, and the fractal analysis was insufficient, we made targeted optimizations based on our "particle-pore-clay synergistic collapsibility mechanism model, including adding correlation and regression analyses between PCAS microparameters and macroscopic collapsibility indicators to clarify the core influencing parameters (macropore ratio, clay content) which are consistent with the conclusions of our model, deepening the fractal analysis by linking the fractal dimension to "microstructure-mechanism-macroscopic deformation", and establishing a logical chain among the three to improve the scientificity of the study.

**Comments 19: Several key conclusions, especially regarding pore transformation, clay migration, and particle contact changes, are presented as deterministic mechanisms, yet the evidential basis is primarily visual observation. Additional explanation or quantitative justification is needed, particularly in Fig. 7–9 discussions. Consider integrating statistical comparisons, parameter references, or reviewing relevant SEM-based literature to reinforce interpretation.**

**Regarding** **Comments 19:** We appreciate the reviewers’ rigorous comments! To address the issue that the demonstration of core mechanisms (pore evolution, clay particle migration, particle contact transformation) relied only on visual micrograph observation, lacking quantitative verification, theoretical interpretation and literature support, we have made systematic supplements to greatly enhance the conclusion persuasiveness. The specific revisions are as follows: Added PCA quantitative analysis and verification of core microscopic parameters: For pore evolution and particle contact transformation, we applied the Principal Component Analysis System (PCAS) to conduct quantitative analysis on key microstructural parameters (see Table 2) to verify core conclusions with quantitative data. The analysis shows that the three loess types had similar pore area ratios before collapse, which decreased by 35.36%, 33.11% and 32.12% for sandy, silty and clayey loess after collapse, respectively. Undisturbed loess featured the highest mesopore ratio and the lowest macropore ratio; after collapse, the mesopore ratio increased by about 8%, the macropore ratio dropped by over 80%, and the micropore ratio decreased slightly. In addition, the pore shape factor increased slightly, probability entropy decreased, pore abundance rose, and pore curvature coefficient increased significantly. These quantitative parameter changes accurately confirm the loess pore system reconstruction law during collapse (collapsible macropores transforming into mesopores, pore morphology becoming regular and simple), directly reflecting the core mechanism of particle rearrangement and soil compaction. This verifies the core conclusions quantitatively and makes up for the deficiency of only visual description via micrographs.

**Comments 20: Sections currently emphasize descriptive repetition rather than analytical synthesis. The authors should expand comparative discussion with prior studies (especially recent SEM/PCAS/fractal works) to demonstrate how their results align with or differ from established knowledge. Conclusions should be more concise, highlight the main contributions, acknowledge limitations, and avoid repeating results verbatim.**

**Regarding** **Comments 20:** Thank you for the reviewer's valuable comments! In response to your requirements that "some chapters focus on descriptive repetition and lack analytical summaries, the authors are advised to expand the comparative discussion with previous studies (especially recent achievements related to SEM/PCAS/fractal theory) to clarify the similarities and differences between the results of this study and existing knowledge, and the Conclusions section should be more concise, highlight core contributions, objectively acknowledge research limitations, and avoid simply repeating test results", we have fully implemented the improvements, and the specific explanations are as follows:

Regarding the requirement of expanding the comparison with previous studies and reducing descriptive repetition, we have completed systematic improvements with reference to the relevant suggestions of Reviewer 1: we have focused on expanding the comparative discussion with previous research results related to SEM, PCAS and fractal theory in recent years, and clearly sorted out the similarities and differences between this study and existing knowledge—clarifying the consensus between the two in aspects such as "the microstructure of loess has fractal characteristics, and microstructural parameters are related to macroscopic collapsibility", while highlighting the innovation of this study (breaking through the limitations of previous single-dimensional and single loess type analysis, carrying out particle-pore coupled fractal analysis, and clarifying the intrinsic relationship between the microstructural evolution and collapsibility mechanism of Malan loess with three typical particle sizes), which effectively makes up for the shortcomings of descriptive repetition and lack of analytical summaries in some chapters.

Regarding the requirement of optimizing the Conclusions section, we have streamlined and condensed the Conclusions section with reference to your suggestions: deleted redundant retellings of test data, and focused on highlighting the four core contributions.
