# Supplementary material for: The microscopic mechanism of water immersion and collapsibility in Malan loess with different particle size
Source: PLoS One. 2026 Apr 23;21(4):e0346775. doi: 10.1371/journal.pone.0346775 (PMC13105354; doi:10.1371/journal.pone.0346775)
Supplement: Attachment — Submitted filename: Response_to_Reviewers_auresp_3.docx [file pone.0346775.s005.docx]

**Response to Reviewers**

Hello! We highly appreciate your valuable suggestions for improving the quality of this manuscript. In particular, we are grateful for your detailed and accurate revision comments — they have pointed out the deficiencies in the original manuscript and greatly inspired the authors. We have carefully revised the manuscript in accordance with the review comments, making the overall expression more standardized and the length more reasonable. We kindly request your review!

**Comments 1: First, although the authors mention correlation and regression analyses between microstructural parameters and collapsibility indicators, the statistical presentation remains relatively limited. Providing more detailed descriptions of the regression results (e.g., fitting quality or statistical significance) would help better support the interpretations.**

**Regarding Comments 1:** We thank the reviewer for pointing out the deficiencies in the statistical presentation. Supplementary explanations regarding the regression analysis between microstructure parameters and collapsibility indicators are provided as follows:

A total of three loess types (sandy loess, silty loess, and clayey loess) were adopted in this study, with three parallel samples for each type, resulting in nine valid groups (n=9). Considering the basic sample size requirements for linear regression analysis, direct implementation of multi-parameter comprehensive regression analysis may lead to biased results; thus, excessive statistical inference was not performed.

In future research, we will expand the sample size by adding loess samples from different regions and depths, and conduct multi-parameter regression analysis to quantify the contribution weight of microstructure parameters to loess collapsibility. Based on the combined qualitative and quantitative demonstration and literature support from the existing data, the current conclusions still maintain sufficient scientificity and reliability.

**Comments 2: Second, the discussion of SEM and PCAS results would benefit from a better explanation of how representative images or fields of view were selected, and how many observations were used in the quantitative analysis. Clarifying these points would strengthen the methodological transparency.**

**Regarding Comments 2:** We sincerely appreciate the valuable comments and suggestions provided by the reviewer. To further improve the transparency and rigor of the research methodology, we have supplemented the manuscript with detailed descriptions of the selection criteria for representative SEM fields of view and the number of observations used in the PCAS quantitative analysis.

These additional clarifications enable readers to better understand the procedures of image selection and quantitative analysis, effectively enhance the standardization and transparency of the experimental method, and strengthen the reliability and persuasiveness of the results. We have revised and improved the relevant content in the manuscript according to the reviewer’s comments.

Thank you to all reviewers and the editor for your meticulous review and valuable suggestions! We have revised the manuscript as requested, enhancing its rigor and persuasiveness. We sincerely appreciate your professional guidance and look forward to the manuscript meeting the publication criteria.
